# Activation of OR10A3 by Suberic Acid Promotes Collagen Synthesis in UVB-Irradiated Dermal Fibroblasts via the cAMP-Akt Pathway

**DOI:** 10.3390/cells11243961

**Published:** 2022-12-07

**Authors:** Wesuk Kang, Dabin Choi, Bomin Son, Soyoon Park, Taesun Park

**Affiliations:** Department of Food and Nutrition, BK21 FOUR, Yonsei University, 50 Yonsei-ro, Seodaemun-gu, Seoul 120-749, Republic of Korea

**Keywords:** OR10A3, suberic acid, collagen, olfactory receptor, dermal fibroblast

## Abstract

In recent years, there has been a great deal of interest in the ectopic roles of olfactory receptors (ORs) throughout the human body. Especially, the ectopic function of OR in the skin is one of the most actively researched areas. Suberic acid, a scent compound, was hypothesized to increase collagen synthesis in the ultraviolet B (UVB)-irradiated human dermal fibroblasts (Hs68) through a specific olfactory receptor. Suberic acid ameliorated UVB-induced decreases in collagen production in Hs68 cells. Using in silico docking to predict the binding conformation and affinity of suberic acid to 15 ectopic ORs detectable in Hs68, several ORs were identified as promising candidates. The effect of suberic acid on collagen synthesis in UVB-exposed dermal fibroblasts was nullified only by a reduction in OR10A3 expression via specific siRNA. In addition, using the cells transiently expressing OR10A3, we demonstrated that suberic acid can activate OR10A3 by assessing the downstream effector cAMP response element (CRE) luciferase activity. We examined that the activation of OR10A3 by suberic acid subsequently stimulates collagen synthesis via the downstream cAMP-Akt pathway. The findings support OR10A3 as a promising target for anti-aging treatments of the skin.

## 1. Introduction

Collagen is a family of extracellular matrix molecules with multiple physiological functions, including the regulation of cell growth, migration, and differentiation [1,2,3]. Collagen constitutes the majority of skin and is the most abundant extracellular structural protein in the dermis (80 percent of skin weight). Dermal fibroblasts produce procollagen and release it into the extracellular milieu, where it is converted to collagen [4,5]. Notably, solar ultraviolet (UV) radiation is a major contributor to the inhibition of procollagen formation, which leads to a decrease in collagen; therefore, extrinsic aging is often referred to as photoaging.

Olfactory receptors (ORs) are the biggest subfamily of G-protein-coupled receptors (GPCR), and it was previously believed that they existed exclusively in olfactory tissue [6]. Recent research has shown, however, that ORs are present in a range of tissues (e.g., skin, muscle, prostate, and liver), where they operate as sensitive chemoreceptors that regulate a number of physiological processes. Particularly, skin is one of the most intensively researched tissues regarding OR’s ectopic functions [6,7,8]. For instance, OR10G7 and OR2AT4 are highly expressed in epidermal keratinocytes, and stimulation of these receptors by their ligands (eugenol and sandalwood) mediates inflammatory response and wound healing, respectively [9,10]. In addition, stimulation of melanocytes with the OR51E2 ligand β-ionone inhibited cell proliferation significantly [11]. These results suggest that ectopically expressed ORs merit further investigation.

Suberic acid is a dibasic acid that is crystalline, colorless and has the chemical formula C_8_O_4_H_14_. It is abundant in several plants, including castor, vernonia galamensis, and hibiscus syriacus [12,13]. In addition to being produced by plants, suberic acid is also produced endogenously in the human body. Moreover, patients with several diseases, such as diabetes, fatty acid oxidation disorders, and medium-chain acyl-CoA dehydrogenase deficiency, had significantly higher suberic acid excretion in urine than healthy subjects, suggesting that suberic acid serves a specific function under adverse conditions [14,15,16]. In a previous study, it was shown that dietary suberic acid protects hairless mice against UVB-mediated skin aging by increasing collagen content and collagen synthesis genes, such as collagen type I alpha 1 chain (*COL1A1*) [17].

The molecular target of suberic acid is largely unexplored, but a few studies suggest that the dicarboxylic acid series including suberic acid may interact with the OR in mammalian cells [18,19]. On the basis of the observation that several ORs are also expressed in human dermal fibroblasts, it is hypothesized that suberic acid stimulates collagen synthesis in UVB-irradiated human dermal fibroblasts by means of a novel OR.

## 2. Materials and Methods

### 2.1. Cell Culture

The human dermal fibroblast cell line (Hs68) was acquired from American Type Culture Collection (ATCC; Manassas, VA, USA). The cells were grown in Dulbecco’s modified Eagle’s medium (DMEM; Gibco, Eggenstein, Germany) supplemented with 10% fetal bovine serum (FBS; Gibco), 1% penicillin-streptomycin (Gibco) at 37 °C, and 5% carbon dioxide.

### 2.2. Quantitative Assessment of Procollagen Secretion

The secretion of procollagen in the medium was determined using a commercially available enzyme-linked immunosorbent assay (ELISA) kit (Takara, Shiga, Japan) according to the manufacturer’s instructions. In brief, cells were seeded and grown overnight in a 24-well plate at 75,000 cells/well. The collected medium was centrifuged to remove debris, and then type I procollagen was measured absorbance at 590 nm using an Infinite M200 microplate reader (Tecan, Männedorf, Switzerland). Results of procollagen I secretion were normalized to total cell protein, as measured by the Bradford protein assay method (BioRad, Hercules, CA, USA).

### 2.3. Determination of Cell Viability

For cytotoxicity measurements, cells were plated at a density of 5000 cells/well in a 96-well plate and grown for 24 h. Cells were then exposed to vehicle (dimethyl sulfoxide, DMSO; Sigma-Aldrich, St. Louis, MO, USA) control or 12.5–400 uM suberic acid (Sigma-Aldrich), and incubated for an additional 24 h. Cell viability was determined using the cell proliferation reagent water-soluble tetrazolium salt (WST-8; Sigma-Aldrich), as recommended by the manufacturer.

### 2.4. Next Generation Sequencing (NGS)

To produce mRNA sequencing libraries, the NEBNext Ultra II Directional RNA-Seq Kit (New England BioLabs, Hitchin, UK) was used. Briefly, total RNA was used using the Poly(A) RNA Selection Kit to isolate mRNA (Lexogen, Vienna, Austria). After purification, the mRNA was chemically fragmented into small fragments, which were then employed as a template for cDNA synthesis using reverse transcriptase, followed by short fragment removal from another purification procedure. After end repair, adapter ligation, and the addition of index codes to samples, PCR amplification was conducted. After adapter ligation and PCR amplification, adapters that were not aligned were eliminated twice. The library quality was examined by Agilent 2100 bioanalyzer DNA high sensitivity kit and the library quantitation was measured by StepOne Real-Time PCR System (Life Technologies, Carlsbad, CA, USA). The libraries were paired-end 100 sequenced on a HiSeq X10 sequencing system (Illumina, San Diego, CA, USA).

### 2.5. Molecular Docking Analysis

The structure data file (SDF) format of suberic acid was retrieved from Pubchem [20]. The 3D structure of the target protein is acquired from AlphaFold, which is a DeepMind-developed artificial intelligence system that predicts a protein’s 3D shape from its amino acid sequence [21]. Additionally, docking of suberic acid to the ORs was conducted using CB-Dock molecular docking software [22]. CB-Dock predicts the binding activities of proteins to chemicals and determines the cavity’s center and size. After identifying the coordinates of the docking pocket, molecular docking and conformational scoring were carried out. The lower the scores, the more stable the compound’s binding to the target, which can be used for a preliminary assessment of the binding affinity of the molecule to the receptor.

### 2.6. Measurement of mRNA Expression

Total RNA was isolated from cells using the TRIzol reagent (Invitrogen, Carlsbad, CA, USA) and measured with a NanoDrop spectrophotometer (Tecan). mRNA expression levels were determined by quantitative real-time polymerase chain reaction (qPCR). qPCR was conducted using SsoAdvanced Universal SYBR Green Supermix (BioRad) and the CFX Real-Time System (BioRad), and mRNA levels were normalized to those of the glyceraldehyde 3-phosphate dehydrogenase (*GAPDH)* gene. Relative expression was calculated using the 2^−^*^ΔΔCT^* method. Semi-quantitative PCR was performed using 2X PCR MasterMix (Intron, Seoul, Korea) and the GeneMax thermal cycler (BIOER; Hangzhou, China) to visually identify the OR10A3 gene expressed in human embryonic kidney cells (HEK293T; ATCC). The primer sequences used for quantitative PCR are listed in Table 1.

### 2.7. Small Interfering RNAs (siRNAs) Transfection

Hs68 cells were seeded in 24-well microplates at the density of 75,000 cells/well. After 24 h, the cells were transfected with 100 mM siRNA (Dharmacon, Lafayette, CO, USA) of each OR gene or non-targeting siRNAs (NT) at a final concentration of 100 nM using lipofectamine 3000 reagent (Invitrogen) in Opti-minimal essential medium (Opti-MEM; Thermo Fisher Scientific, Bremen, Germany), as per the manufacturer’s instructions. After incubation for 24 h, the Opti-MEM medium was replaced with the fresh DMEM medium containing 10% FBS, and cells were cultured for another 24 h. The siRNA sequences are provided in Table 2.

### 2.8. Cyclic Adenosine Monophosphate (cAMP) Measurement

After removing the media, the cells were rinsed with phosphate-buffered saline (PBS) and treated for 5 min with 0.1 M hydrochloric acid. The concentrations of cAMP in the collected lysates were then measured using a cAMP ELISA kit (Enzo Life Sciences, Farmingdale, NY, USA) according to the manufacturer’s instructions. Briefly, the lysates were neutralized and the cAMP conjugate was added to the binding sites on an IgG-coated microplate in order to compete with cAMP. The unbound cAMP was then eliminated by three washes with PBS. The substrate was then carefully added to each well in order to determine the enzyme’s bound activity. After stopping the reaction, the relative optical density at 450 nm was estimated using a microplate reader. The cAMP concentration was normalized to the total intracellular protein concentration, which was obtained using the Bradford reagent (Biorad).

### 2.9. Western Blot

The cells were harvested and lysed using a protein extraction solution (PRO-PREP; iNtRON, Seoul, Korea). After centrifugation at 13,000× *g* for 20 min at 3 °C, 25 μg of protein in the lysates were separated on 12.5% polyacrylamide gels. Protein was transferred to nitrocellulose membranes (Whatman, Maidstone, UK) by electrophoresis. Membranes were blocked with 5% BSA in tris-buffered saline containing 0.1% Tween-20 (TBST). Primary antibodies (Cell signaling, Danvers, MA, USA) were incubated with polyvinylidene difluoride membrane for 1 h at room temperature. Blots were washed five times with TBST solution and incubated with an appropriate secondary antibody for 1 h at 20 °C. After washing three times with TBST, the blots were developed with an electrochemiluminescence detection reagent (Biomax, Seoul, Korea) and were scanned by using Light-capture (ATTO, Tokyo, Japan). The intensities of specific bands were quantified and normalized using GAPDH as a marker for loading control.

### 2.10. Plasmid Construct, Transfection, and Dual Luciferase Reporter Assay

The human gene encoding OR10A3 was purchased from Novoprolabs (Cat# 746761-1; Shanghai, China) and subcloned into the pcDNA3.1 vector (Promega, Madison, WI, USA). Then, HEK293T cells were seeded into a 24-well plate (100,000 cells/well) and cultured for 24 h. The recombinant plasmid containing the OR10A3 was subsequently co-transfected into the HEK293 cells with (1) Renilla luciferase reporter plasmid (pRL-TK; Promega, Madison, WI, USA), (2) cAMP response element firefly luciferase construct (pCRE-luc; Promega), and (3) accessory proteins that facilitate the translocation of the olfactory receptor to the cell membrane (RTP1S, Ric8B, and Golf; gifts from Hiroaki Matsunami at the University of Duke in the USA and Cheil Moon at Daegu Gyeongbuk Institute of Science and Technology (DGIST) in Korea), using lipofectamine 3000 transfection reagent (Thermo Fisher Scientific). 48 h after transfection, cells were treated with vehicle control or suberic acid (100 uM), incubated for further 6 h, and harvested for dual-luciferase assay using a Dual-Glo Luciferase assay system (Promega). The firefly luciferase signal was measured first and followed by Renilla luciferase in the same sample using a GloMax 20/20 luminometer (Promega).

### 2.11. Statistical Analysis

The Student’s *t*-test was used to assess the significance of the differences between the two groups. SPSS 25 was used to conduct statistical analysis (Chicago, IL, USA). In all statistical tests, differences with *p* values of 0.05 or less were regarded as statistically significant.

## 3. Results

### 3.1. Ultraviolet B (UVB) Inhibits Procollagen Synthesis in Hs68 Cells

**Figure 1:**
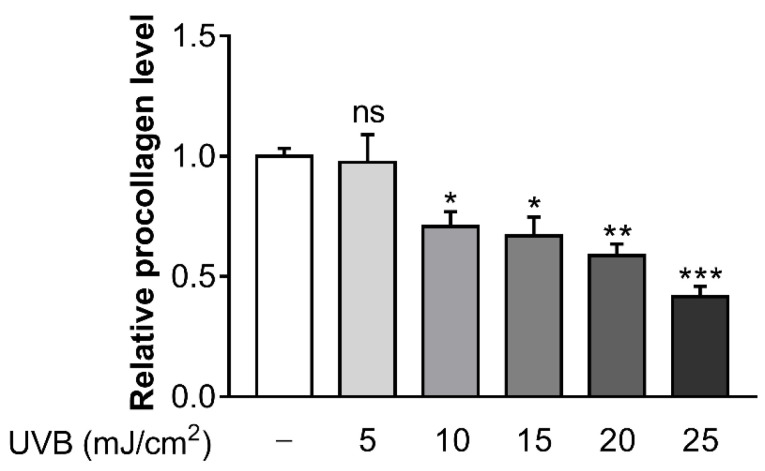
Ultraviolet B (UVB) inhibits procollagen synthesis in Hs68 cells. The cells were irradiated for 24 h with various doses of UVB (5, 10, 15, 20 and 25 µM). Procollagen concentration was determined by enzyme linked immunosorbent assay (ELISA) assay. Data are presented as mean ± standard error of the mean (SEM) of three independent experiments. Statistically significant differences are marked as * *p* < 0.05, ** *p* < 0.01, *** *p* < 0.001; ns, not significant.

To determine the optimal UVB dose which caused a reduction in collagen synthesis, several doses of UVB (5, 10, 15, 20, and 25 mJ/cm^2^) were irradiated in Hs68 cells. At UVB doses of 10 mJ/cm^2^, procollagen concentration began to decrease significantly (Figure 1). Consequently, this UVB dose was chosen as the suitable UVB dosage for further experiments in the current study.

### 3.2. Suberic Acid Attenuated the Reduction of Collagen Production in UVB-Irradiated Hs68 Cells

**Figure 2:**
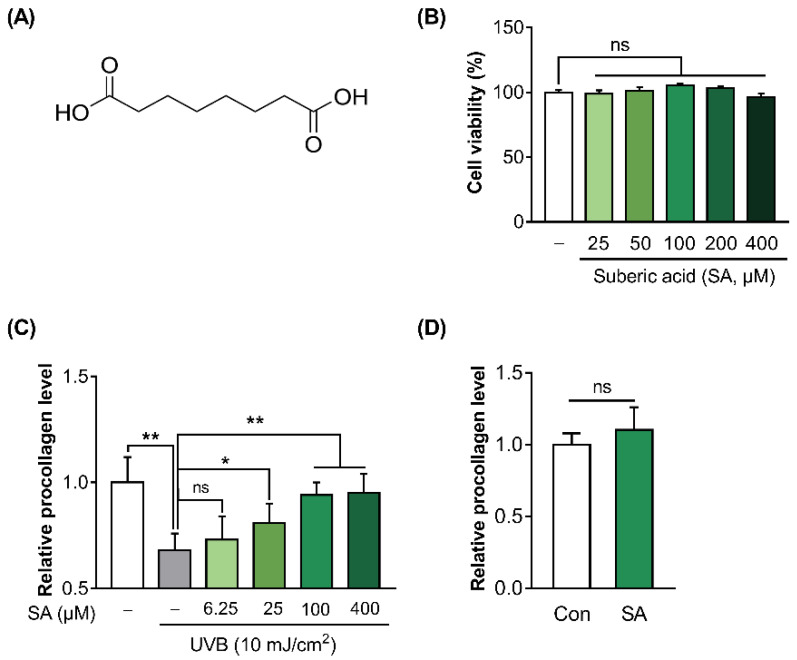
Suberic acid attenuated the reduction of collagen production in UVB-irradiated Hs68 cells. (**A**) The molecular structure of suberic acid. (**B**) Cell viability of Hs68 cells treated with various concentrations of suberic acid (SA; 25–400 μM) for 48 h. (**C**) Procollagen concentrations in the supernatant of Hs68 cells treated with various doses of suberic acid (6.25–400 μM) for 48 h after 10 mJ/cm^2^ UVB exposure. (**D**) Hs68 cells were treated with vehicle or 100 μM suberic acid for 48 h in the absence of UVB irradiation and the concentrations of procollagen in the supernatant were evaluated. The results are shown as means SEM (*n* = 3). Significant differences between groups are indicated by * *p* < 0.05; ** *p* < 0.01; ns, not significant.

The molecular structure of suberic acid is presented in Figure 2A. To test the effect of suberic acid on the cell viability of Hs68, WST-8 assay was conducted. Suberic acid treatment until 400 μM did not show any cytotoxicity compared with vehicle (DMSO)-treated control (Figure 2B). In addition, to investigate the effect of suberic acid on collagen production in UVB-treated Hs68 cells, the procollagen concentration in the culture supernatants of Hs68 cells was quantified. UVB exposure significantly decreased procollagen production in Hs68 cells. In contrast, suberic acid increased the production of procollagen in a dose dependent manner (Figure 2C). Since the effect of suberic acid on the collagen synthesis seemed to saturate at around 100 uM, all further experiments were performed with 100 uM suberic acid concentration. Additionally, treatment with suberic acid (100 uM) did not result in an increase in the synthesis of procollagen in control cells that had not been exposed to UVB. (Figure 2D).

### 3.3. Suberic Acid Increased the Production of Collagen through OR10A3 in UVB-Exposed Hs68 Cells

It was hypothesized that the ORs could be a target of suberic acid. We analyzed the expression profiles (FPKM) of OR genes in Hs68 cells based on NGS sequencing data and found that at least 15 OR were clearly expressed in Hs68 cells (FPKM > 0.01). Then, we conducted a virtual screening of suberic acid-ORs binding affinities using autodock vina program. As a result, the top five ORs, OR1L8, OR2H2, OR10A3, OR10A4, and OR10A6, were selected (Table 3).

**Figure 3:**
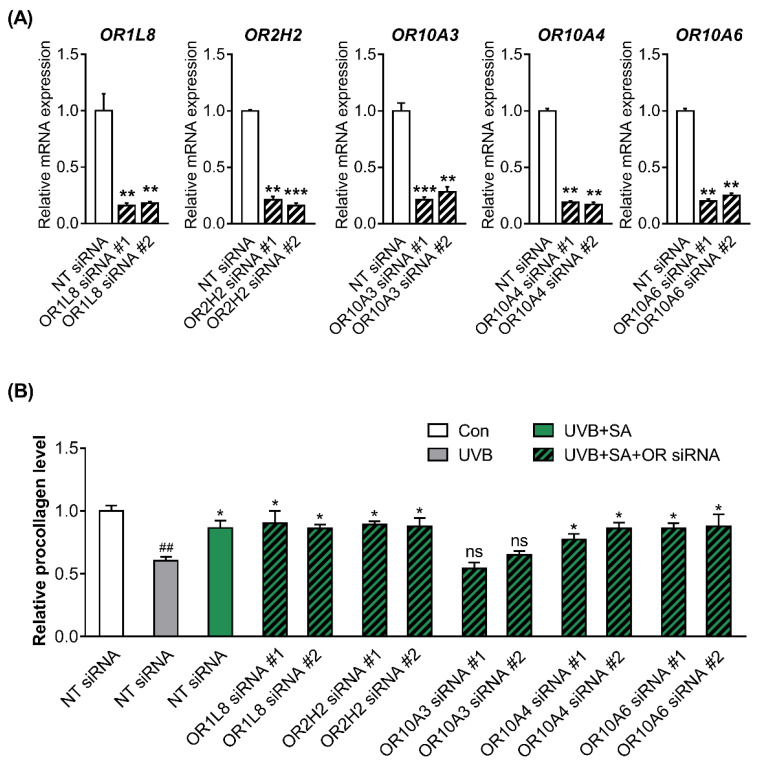
Suberic acid increased the production of collagen through OR10A3 in UVB-exposed Hs68 cells. (**A**) The cells were treated with or without siRNA against 5 ORs (OR1L8, OR2H2, OR10A3, OR10A4 and OR10A6). After treatment for 24 h, the relative mRNA expression of 5 ORs was analyzed. Significant differences between groups are shown as ** *p* < 0.01; *** *p* < 0.001 vs. NT siRNA. (**B**) Hs68 cells were treated with suberic acid (SA; 100 uM) or the vehicle after being exposed to UVB. The siRNA against 5 ORs or NT siRNA were pre-treated for 1 h before the suberic acid treatment. Procollagen contents in the supernatant were analyzed. Significant differences between groups are shown as * *p* < 0.05 vs. Con; ## *p* < 0.01 vs. UVB; ns, not significant. Results are shown as means ± SEM (*n* = 3).

To confirm the knockdown efficiency, two siRNAs (siRNA #1 and siRNA #2) targeting different regions in the respective five ORs were designed, and the ORs expression was measured in Hs68 cells transfected with NT, OR siRNA #1 or #2. All siRNAs used in the present study achieved over 80% reduction of each gene expression (Figure 3A).

To determine whether ORs are involved in the formation of collagen by suberic acid treatment, the procollagen concentrations in the culture supernatants of UVB-exposed Hs68 cells transfected with NT siRNA or two different siRNAs #1 and #2 against 5 ORs were measured after treatment with suberic acid (100 uM). Notably, the effect of suberic acid on collagen production was abolished only in OR10A3 siRNA-transfected cells (Figure 3B).

### 3.4. The Treatment with Suberic Acid Led to the Activation of OR10A3

**Figure 4:**
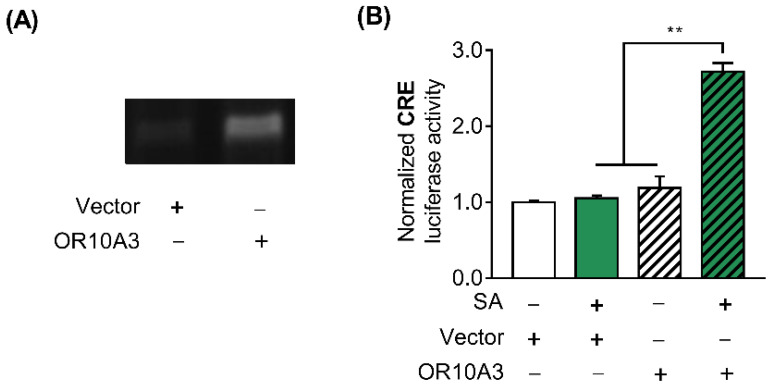
The treatment with suberic acid led to the activation of OR10A3. (**A**) Control plasmid vector or plasmid harboring OR10A3 were introduced into HEK293T cells. The cells were harvested after 48 h and the overexpression of OR10A3 was verified by a semi-quantitative PCR in OR10A3-transfected HEK293T cells. (**B**) HEK293T cells were co-transfected with the plasmids encoding OR10A3, RTP1S, Golf, and Ric8b, pCRE-luc, and pRL-TK for 48 h. Then, the cells were incubated with vehicle control or suberic acid (SA; 100 uM) for 6 h, and CRE promoter activity was analyzed using the dual-luciferase assay. The results are expressed as the mean SEM of three independent experiments. ** *p* < 0.01.

After establishing that suberic acid’s effect on collagen production is mediated by OR10A3, we sought to confirm that suberic acid directly activates OR10A3 and modulates downstream signaling. To test this hypothesis, HEK293T cells were transfected with the plasmids encoding the olfactory receptor OR10A3 and the accessory proteins (RTP1S, Golf, and Ric8b) that aid in transporting the olfactory receptor to the cell membrane, pCRE-luc, and pRL-TK. We first verified by a semi-quantitative RT-PCR that the overexpression of the OR10A3 gene was achieved in the HEK293T cell line (Figure 4A). Since it is well known that the cAMP/PKA/CREB signaling cascade is activated when a scent compound binds to an olfactory receptor, the activity of the CRE promoter in response to suberic acid treatment was assessed to determine whether suberic acid indeed binds to and activates OR10A3 [23]. In the presence of suberic acid (100 uM; the concentration of significant collagen stimulating effect), HEK293T cells transfected with a plasmid encoding the OR10A3 showed a significant increase in CRE-luciferase activity than those transfected with the empty vector (Figure 4B).

### 3.5. The Activation of OR10A3 by Suberic Acid Increased the Production of Collagen via Downstream cAMP in UVB-Exposed Hs68 Cells

**Figure 5:**
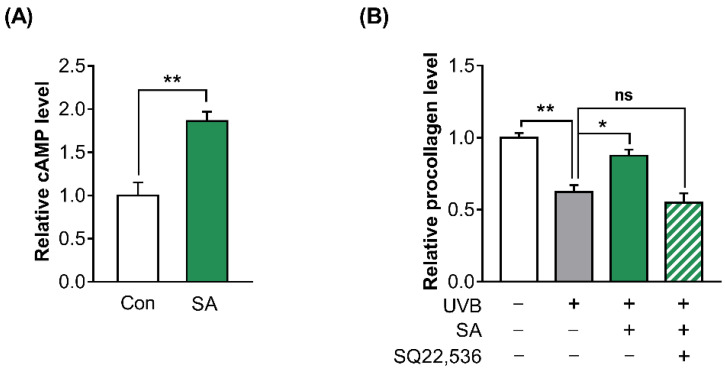
The activation of OR10A3 by suberic acid increased the production of collagen via downstream cAMP in UVB-exposed Hs68 cells. (**A**) The cells were treated with suberic acid (SA; 100 uM) or the vehicle and then intracellular cAMP levels were analyzed. (**B**) Procollagens concentrations in the supernatant of UVB-exposed Hs68 cells treated with suberic acid (100 uM) and SQ22,536 (50 uM) for 24 h. Results are shown as means ± SEM (*n* = 3). Significant differences between groups are shown as * *p* < 0.05; ** *p* < 0.01; ns, not significant.

Due to the fact that OR10A3 belongs to the G protein-coupled receptor superfamily, the levels of downstream intracellular cAMP in Hs68 cells were analyzed. Indeed, treatment with suberic acid (100 uM) resulted in a significant increase in cAMP levels (Figure 5A).

To investigate whether elevated intracellular cAMP concentration by suberic acid mediates collagen production in UVB-exposed Hs68 cells, the procollagen concentration was analyzed in UVB-exposed Hs68 cells after treatment with suberic acid and the cAMP inhibitor (SQ22,536). The treatment with SQ22,536 (50 uM) completely blocked the effects of suberic acid on procollagen production (Figure 5B).

### 3.6. Suberic Acid Increased Collagen Production in UVB-Exposed Hs68 Cells through the Akt-Dependent Signaling Pathway

To study the suberic acid-mediated downstream effector of the cAMP pathway, we queried the pattern of gene expression alterations following suberic acid treatment in the NGS dataset using the connectivity map bioinformatics platform. Notably, the pattern of gene expression altered by suberic acid treatment showed the greatest negative correlation with the pattern of phosphatidylinositol-3-kinase (PI3K) inhibitor treatment, suggesting that suberic acid may serve as a PI3K activator (Table 4).

**Figure 6:**
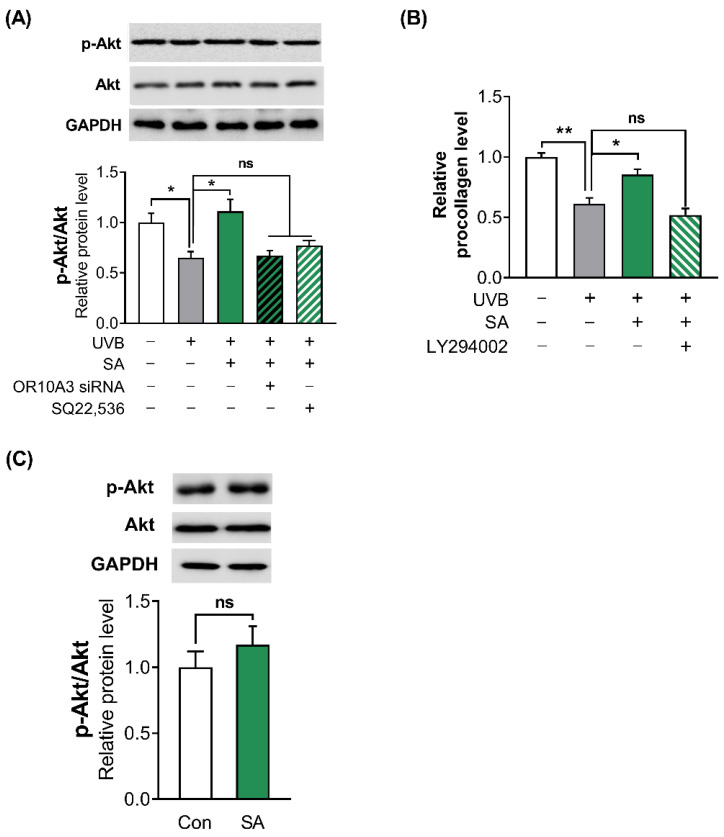
Suberic acid increased collagen production in UVB-exposed Hs68 cells through the Akt-dependent signaling pathway. Hs68 cells were treated with suberic acid or the vehicle after being exposed to UVB. (**A**) The OR10A3 siRNA or SQ22,536 (50 uM) was pre-treated for 1 h before the suberic acid treatment (SA; 100 uM). Then, Akt phosphorylation was analyzed. (**B**) The LY294002 (50 uM) was pre-treated for 1 h before the suberic acid treatment. Procollagen contents in the supernatant were analyzed. (**C**) Suberic acid-mediated Akt phosphorylation was analyzed without UVB exposure. Glyceraldehyde-3-phosphate dehydrogenase (GAPDH) was used as an internal control. Data are presented as mean ± SEM of three independent experiments. Statistically significant differences are marked as * *p* < 0.05, ** *p* < 0.01; ns, not significant.

To confirm whether suberic acid treatment activates PI3K, the phosphorylation of its downstream effector Akt was analyzed in UVB-exposed Hs68 cells after treatment with suberic acid, OR10A3 siRNA and SQ22,536. Suberic acid treatment significantly increased phosphorylation of Akt in UVB-exposed Hs68 cells. In contrast, each OR10A3 siRNA and SQ22,536 treatment (50 uM) attenuated the effects of suberic acid on Akt phosphorylation (Figure 6A). Furthermore, the PI3K inhibitor (LY294002) treatment (50 uM) nullified the effects of suberic acid on procollagen production (Figure 6B). Interestingly, suberic acid treatment did not increase the phosphorylation of Akt in UVB-unexposed control cells (Figure 6C).

### 3.7. The Activation of OR10A3 by Suberic Acid Enhanced Gene Expression of Akt Downstream Transcription Factor SP1 and Its Target Gene COL1A1 in UVB-Exposed Hs68 Cells

**Figure 7:**
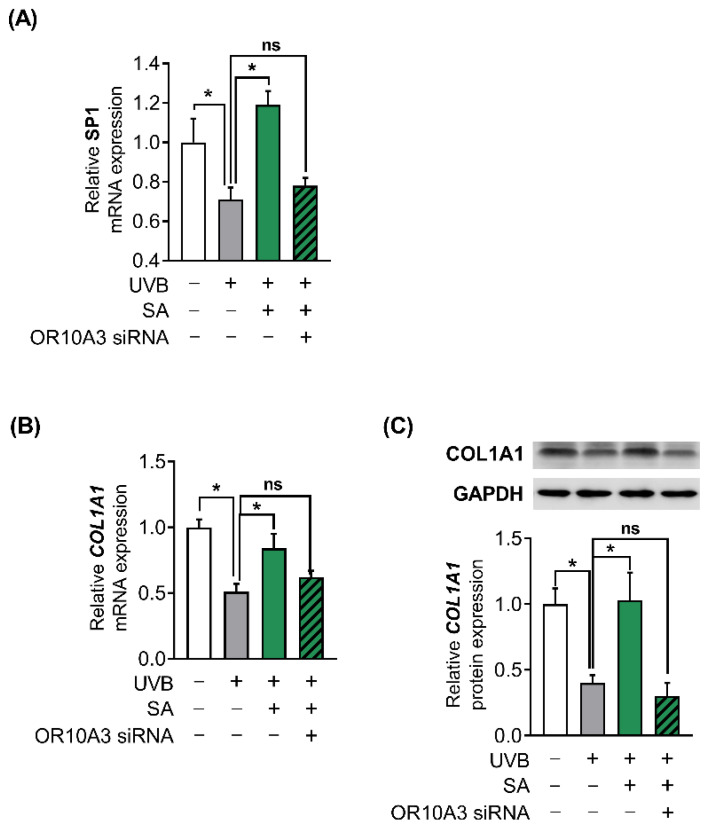
The activation of OR10A3 by suberic acid enhanced gene expression of Akt downstream transcription factor and its target gene COL1A1 in UVB-exposed Hs68 cells. The cells were treated with suberic acid (SA; 100 uM) or the vehicle after being exposed to UVB. The OR10A3 siRNA or NT siRNA was pretreated for 1 h before the suberic acid treatment. (**A**) The mRNA expression of SP1 was analyzed. (**B**,**C**) The mRNA and the protein expression of COL1A1 were analyzed. GAPDH was used as an internal control. Data are presented as mean ± SEM of three independent experiments. Statistically significant differences are marked as * *p* < 0.05; ns, not significant.

**Figure 8:**
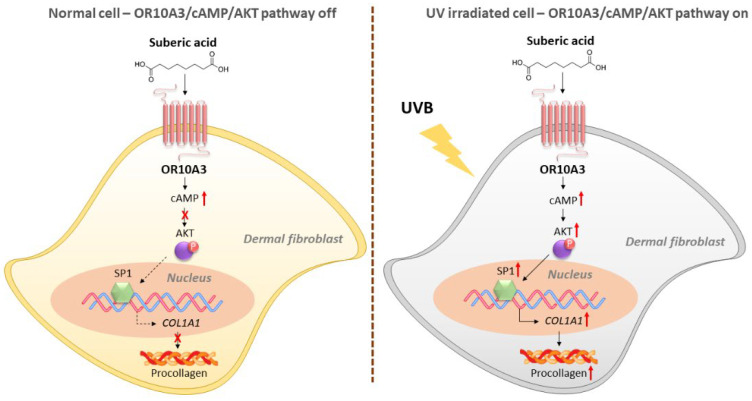
A schematic diagram illustrating the proposed mechanism by which suberic acid increases collagen through OR10A3/cAMP/Akt-dependent signaling pathway in UV-exposed dermal fibroblasts.

To investigate how the Akt signaling pathway contributes to the production of collagen, the gene expression of Akt downstream transcription factor, SP1 and its target gene, COL1A1 was measured. Suberic acid treatment (100 uM) enhanced gene expression of SP1 in UVB-exposed Hs68 cells, in contrast, OR10A3 siRNA treatment abolished the effects of suberic acid on SP1 gene expression (Figure 7A). Likewise, COL1A1 mRNA expression was elevated in Hs68 cells treated with suberic acid compared to those treated with vehicle. The stimulatory effect of suberic acid on COL1A1 mRNA was completely blocked by OR10A3 siRNA (Figure 7B). We further verified that suberic acid increased COL1A1 levels through OR10A3 at the protein level (Figure 7C). Taken together, the activation of the OR10A3-cAMP-Akt pathway is a likely mechanism contributing to collagen synthesis effects of suberic acid in UVB-exposed dermal fibroblasts (Figure 8).

## 4. Discussion

G-protein-coupled receptors have long been of interest as molecular targets because they regulate diverse physiological processes and have surface-accessible binding sites; more than 30 percent of US Food and Drug Administration-approved drugs target GPCRs [24,25,26,27]. Notably, ORs account for nearly half of the GPCRs in humans; however, these ORs were largely excluded as potential molecular targets for human therapy because they were believed to be solely associated with smell-related functions. Currently, ORs are thought to be intimately involved in a variety of physiological processes and could therefore represent a promising therapeutic target class; however, their application remains challenging. Factors contributing to the limited targeting of ORs include the fact that 90% of orphan ORs lack ‘tool compounds’ to aid in defining their functional roles, as well as an incomplete understanding of signaling mechanisms [6,28,29]. This study deorphanized OR10A3 with a natural ligand (suberic acid) and identified a related downstream signaling pathway, allowing the functional characterization of OR10A3 in human dermal fibroblasts.

Even though ORs are ectopically produced in a variety of non-olfactory organs, doubts have often been raised concerning the potential functions of the ectopic receptors owing to the typically low levels of the transcripts [7]. Notably, the Human Protein Atlas, a public database of protein expression profiles, indicates that OR10A3 is highly expressed in two tissues, the skin and brain (cerebral cortex, cerebellum, etc.) [30]. The current data provide additional support for the hypothesis that the OR expression profile in human tissues provides insight into OR’s unknown functions. In the future, it will be intriguing to explore the possible functions of OR10A3 in the brain.

cAMP is a second messenger that regulates a wide variety of intracellular processes in biological systems. It has been reported that cAMP signaling controls various downstream signaling pathways, both directly and indirectly, depending on the situation, such as the type of upstream GPCR and cell status; the complex nature of cAMP makes it difficult to investigate the cAMP-triggered downstream events [31,32,33,34,35]. To address this issue, this study utilized the connectivity map [36], a bioinformatics tool that searches for similarities between a target signature and a reference collection of expression profiles, and discovered that OR10A3-induced cAMP signaling activates the well-known Akt pathway for collagen synthesis. In accordance with the findings of this study, the Akt pathway has frequently been implicated in OR/cAMP-mediated signaling pathways. A synthetic sandalwood odorant, for example, increases human hair growth by activating the OR2AT4/cAMP/Akt pathway [10]. Furthermore, activation of OR10J5 in response to odorant lyral promotes angiogenesis by increasing cAMP levels and Akt phosphorylation [37].

Collagen in the skin is essential not only for cosmetic purposes, including wrinkles, flexibility, etc. but also for pathological events. Specifically, collagen is recognized as a crucial role in wound healing, since collagen, a fundamental component of the extracellular matrix, plays a pivotal role in the control of the stages of wound healing either in its native, fibrillar conformation or as soluble components in the wound environment. Impairments in any of these stages result in a chronic, nonhealing wound that, in most cases, requires some type of intervention to restore the process to its normal condition. Collagen, which is central to the control of these processes, has been exploited as an adjunct to wound therapy to promote healing [38,39]. Unfortunately, the role of OR10A3 in the wound could not be confirmed in the current in vitro system. Further studies using in vivo models would warrant extending the beneficial effects of OR10A3 on wound healing.

After examining the function of OR10A3 at the in vitro level, the authors attempted to explore its function in an in vivo model, which could be more physiologically relevant than the culture of specific cell types or cell lines. Several studies on the ectopic function of OR have used specific gene knockout mice; however, it is not a simple task to identify the murine ORs corresponding to the human genes. Since the OR gene family is extremely large and their number varies by species (e.g., 1000 members in mice and 400 members in humans), the murine ortholog for specific human OR is frequently unclear [40]; it is even doubtful that one-to-one ortholog pairs exist between human OR and mouse OR. In the case of OR10A3, at least five mouse genes (olfr512, 514, 516, 517, 518, and 519) show more than 75% similarity to the human OR10A3 gene [41], implying that one of those murine genes is the true OR10A3 ortholog, or those genes may have a one-to-many or many-to-many ortholog relationship with OR10A3. This complex issue will be further investigated in the future.

## 5. Conclusions

In summary, the present study revealed that human dermal fibroblasts express OR10A3, a subtype of GPCR. The activation of OR10A3 by suberic acid increases the cAMP/Akt signaling pathway, which contributes to the increased production of collagen in dermal fibroblasts exposed to ultraviolet light. To our knowledge, this is the first report of an ectopic OR playing a significant role in collagen synthesis. These results suggest that OR10A3 could be a therapeutic target for anti-aging of the skin.

## Figures and Tables

**Table 1:** Primer sequences used for RT-PCR.

Gene Description	Sequences (5′→3)
Collagen type I alpha 1 chain (*COL1A1*)	F: ACATGTTCAGCTTTGTGGACC
	R: TGTACGCAGGTGATTGGTGG
Olfactory receptor family 1 subfamily L member 8 (*OR1L8*)	F: GCCCTGTGCTGAAATTGTCC
	R: GGCTTTGCGTTTCCCAGAAG
*OR2H2*	F: CCATCTCACTGTGGTCACCCTCTTC
	R: GAATGCCCTGGTTACCTCCTTGTTC
*OR10A3*	F: ATCTGGCTACTCACCCGAAAC
	R: AGATGAGCGGATTGAGCAGAG
*OR10A4*	F: CACCTCTTGGTTGTCTCTCTCTTC
	R: CCTTCAGTTTCCATCTAAGCCAATC
*OR10A6*	F: GTCAACAGAGAAAGGTTCGGG
	R: TGGTGTCTTGGATAGATCACTG
Glyceraldehyde-3-phosphate dehydrogenase (*GAPDH*)	F: GACAGTCAGCCGCATCTTCT
	R: CGCCCAATACGACCAAATC

**Table 2:** siRNA sequences used for gene silencing.

Gene Name	siRNA Sequence (Sense [S], Antisense [A])	
*OR1L8*	#1	S: CGUCUCACCUUCUGUGACU A: AGUCACAGAAGGUGAGACG
	#2	S: CCUACGCUGUCAAGGACCA A: UGGUCCUUGACAGCGUAGG
*OR2H2*	#1	S: CUGUUUCACCACGAGUUGU A: ACAACUCGUGGUGAAACAG
	#2	S: GUCAUUGGGCUAGUGGAGU A: ACUCCACUAGCCCAAUGAC
*OR10A3*	#1	S: CUCUGAACUACCCAGUGAU A: AUCACUGGGUAGUUCAGAG
	#2	S: CUCAUCUAUAGCUUACGAA A: UUCGUAAGCUAUAGAUGAG
*OR10A4*	#1	S: CUGAUCAUUCAAGACACAA A: UUGUGUCUUGAAUGAUCAG
	#2	S: CUCUCUCUUCUAUAGCACU A: AGUGCUAUAGAAGAGAGAG
*OR10A6*	#1	S: CUGAAUCUGCUUAUCUACA A: UGUAGAUAAGCAGAUUCAG
	#2	S: CAUCCUCUCAACUACCAAA A: UUUGGUAGUUGAGAGGAUG
Non-targeting siRNA (NT)	S: GAACUGAUGACAGGGAGGC A: GCCUCCCUGUCAUCAGUUC	

**Table 3:** The expression profiles (FPKM) of 15 candidates olfactory receptor (OR) genes detectable in Hs68 cells and virtual screening of suberic acid-ORs binding affinities using autodock vina program.

Olfactory Receptor (OR) Name	FPKM	Vina Score
**OR1L8**	0.035	**−5.5**
OR2A1/42	0.192	−4.8
OR2A4/7	0.413	−4.9
OR2AE1	0.254	−4.4
OR2B2	0.070	−4.7
**OR2H2**	0.029	**−5.7**
OR6A2	0.041	−5.2
**OR10A3**	0.035	**−5.7**
**OR10A4**	0.032	**−5.8**
OR10A5	0.017	−5.1
**OR10A6**	0.034	**−5.6**
OR51B5	0.063	−4.9
OR51I1	0.581	−4.5
OR52DI	0.274	−4.6
OR56B4	0.026	−5.2

**Table 4:** Connectivity results between suberic acid-modulated genes in Hs68 cells and compounds in the connectivity map platform.

Name	Description	Score
**BRD-75430629**	**PI3K Inhibitor**	**−97.92**
Etilefrine	Adrenergic recetptor agonist	94.47
2-(4-methoxybenzylthio)-6-methylpyrimidin-4-ol	Matrix metalloproteinase inhibitor	93.53
Iloprost	Prostanoid receptor agonist	91.53
UNC-0321	Histone lysine methyltransferase inhibitor	−90.10

## Data Availability

The data that support the findings of this study are available from the corresponding author upon reasonable request.
